# Mapping the Potential Distribution of Major Tick Species in China

**DOI:** 10.3390/ijerph17145145

**Published:** 2020-07-16

**Authors:** Xin Yang, Zheng Gao, Tianli Zhou, Jian Zhang, Luqi Wang, Lingjun Xiao, Hongjuan Wu, Sen Li

**Affiliations:** 1 College of Environment Science and engineering, Huazhong University of Science and Technology, Wuhan 430070, China; yang_xin@hust.edu.cn (X.Y.); hggz@hust.edu.cn (Z.G.); wang_luqi@hust.edu.cn (L.W.); xiaolingjun@hust.edu.cn (L.X.); hongjuanwu@mail.hust.edu.cn (H.W.); 2 School of Automation, Wuhan University of Technology, Wuhan 430070, China; zhoutianli@whut.edu.cn (T.Z.); jian_zhang@whut.edu.cn (J.Z.); 3 UK Centre for Ecology & Hydrology, Wallingford OX10 8BB, UK; 4 Environmental Change Institute, University of Oxford, Oxford OX1 3QY, UK

**Keywords:** tick, potential distribution, environmental factors, MaxEnt, machine learning

## Abstract

Ticks are known as the vectors of various zoonotic diseases such as Lyme borreliosis and tick-borne encephalitis. Though their occurrences are increasingly reported in some parts of China, our understanding of the pattern and determinants of ticks’ potential distribution over the country remain limited. In this study, we took advantage of the recently compiled spatial dataset of distribution and diversity of ticks in China, analyzed the environmental determinants of ten frequently reported tick species and mapped the spatial distribution of these species over the country using the MaxEnt model. We found that presence of urban fabric, cropland, and forest in a place are key determents of tick occurrence, suggesting ticks were likely inhabited close to where people live. Besides, precipitation in the driest month was found to have a relatively high contribution in mapping tick distribution. The model projected that theses ticks could be widely distributed in the Northwest, Central North, Northeast, and South China. Our results added new evidence on the potential distribution of a variety of major tick species in China and pinpointed areas with a high potential risk of tick bites and tick-borne diseases for raising public health awareness and prevention responses.

## 1. Introduction

Ticks are widely distributed across the world and considered to be the second most dangerous carriers of disease causative agents next to mosquito [1,2]. Through tick bites, many of these pathogens could be transmitted to animals and, accidentally, humans. For example, the tick-borne encephalitis virus had caused widespread infections in East Europe, West Europe, Middle Europe, and Russia [3,4]. Since ticks can inhabit a wide range of vegetated habitats in the countryside, suburbs or even urban areas [5,6,7], they are posing threats to public and veterinary health wherever their population is established.

Understanding the environmental determinants and spatial pattern of ticks’ distribution is fundamental to the prevention and control of tick-borne diseases. Existing studies on the ecological distribution of ticks across the temperate Northern Hemisphere are numerous [8,9,10], and have identified a range of environmental factors associated with tick occurrence, including air humidity, precipitation, temperature, soil humidity, vegetation type, land use, and interference [11,12,13,14,15]. Lindgren et al. [16] and Danielová et al. [17] found that the latitudinal and altitudinal limits of tick distribution have changed as the climate warms. Evaluating how these environmental factors could shape the geographic distribution and population dynamics of ticks became one of the most important research directions in the field of ticks and tick-borne diseases.

Models are commonly used in examining the existing spatial distribution of tick species and projecting the potential distribution in places where data are scarce or investigations on tick’s occurrence have not been conducted. These research goals could generally be accomplished by using either ecological process-based tick population models [18,19,20] or species distribution models (SDMs) based on data associations [21,22]. Species distribution models are frequently used in predicting the spatial distribution of disease vectors such as mosquitoes and sandflies [23,24]. The maximum entropy model (MaxEnt) is an artificial intelligence model based on machine learning techniques. Its predictive performance is considered amongst the highest levels of existing SDM methods [25]. Since it is particularly useful for presence-only data, it has been widely used in predicting the potential distribution of invasive species, the planning of species reserves, and the response to spatial distribution of species to climate change [26].

Previous SDM studies on tick distribution mainly used climatic variables (temperature, precipitation, etc.) to predict the climate suitability for tick occurrence [27,28,29]. However, the spatial pattern of tick habitats has also known to be affected through a series of anthropogenic and environmental factors and processes related to land-use/land-cover change (LUCC) [30]. At the local level, LUCC could alter not only local surface conditions but also change microclimatic conditions [31], which could further affect the habitat suitability of ticks at various extents. On the global scale, surface vegetation change may gradually impact on global atmospheric circulation which could, in turn, alter the climatic conditions at lower levels [32,33]. Moreover, the construction of impervious infrastructure (such as buildings and roads) that replaced (semi-)natural open land could destroy the habitats of ticks and their host animals [34]. With the development of remote sensing techniques, the spatio-temporal dynamics of LUCC could be better monitored and characterized for a more comprehensive quantification of a species–response curve along the environmental gradient, contributing to an improved predictability power of SDM [35].

However, existing studies on tick distribution at the national level remain rare in China where ticks were found to be widely distributed, and the public awareness on tick-borne diseases are limited [36,37]. With the implementation of large-scale afforestation strategy in China, the extent of forested areas in China has shown a rapid growth trend [38]. This potentially enlarges the distribution of ticks, as forests are known as ticks’ most favorite habitat type [39,40]. Meanwhile, the expanding eco-tourism industry and rising number of outdoor leisure pursuers in China [41] could infer increased human visitation in tick-infested areas and, thus, increased tick–human contact rate. Provided such a situation, studies on ticks and tick-borne diseases recently gained attention, and there has been an increasing number of studies on reporting cases of tick-borne infections in humans or livestock, and the clinical treatment of tick-borne diseases [42,43]. Nevertheless, existing shreds of evidence on the wide range of ticks’ major host animals in China have been extensively summarized in several recent review articles [44,45]. Knowledge of the spatial pattern and underpinning factors of tick distribution remain very limited.

The main objectives of this study were to: (i) identify key environmental factors on the known spatial distribution of ticks in China and (ii) map the potential spatial distribution model of major species of ticks in China. We made use of the recently compiled information on the known spatial distribution samples of major species of ticks in China [42,43]. We then collected and prepared bioclimatic, soil, and land-use factors from various sources as the potential determinants of tick distribution. By using a set of different machine learning techniques, we analyzed the effects of these factors on the occurrence of ticks. Finally, the MaxEnt model was used to project the potential spatial distribution of major tick species across China.

## 2. Materials and Methods

### 2.1. Processing Tick Occurrence Data

**Figure 1:**
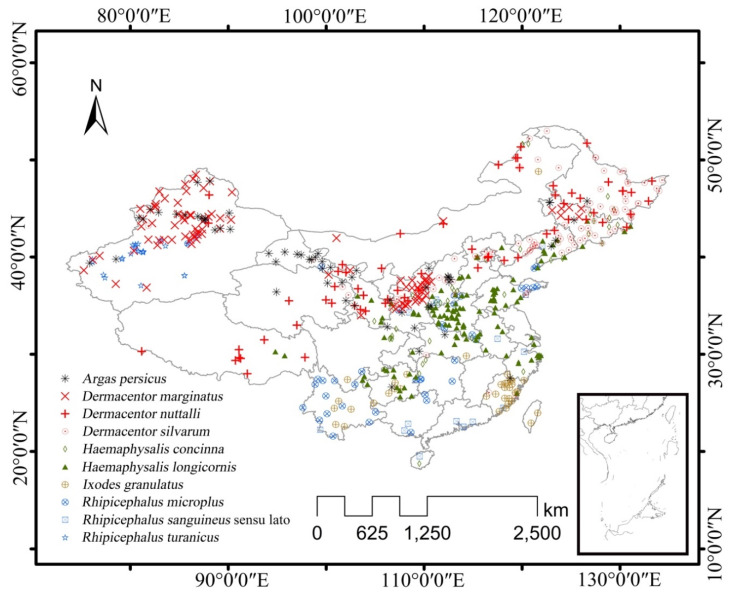
Distribution of selected major tick species in China.

In our previous study, a spatial dataset of 123 tick species distributed over 1100 locations was compiled based on peer-reviewed Chinese and English literature published between 1960 and 2017 [43]. In order to select suitable tick occurrence data, we kept the data at lower (i.e., prefectural, county, township, and finer) levels. The remaining occurrence data were refined using the Spatial Distribution Modeling toolbox (SDMToolbox 1.1c) [27]. We then removed duplicate points with identical coordinates, leaving 976 (out of 5731) data points for the modelling exercise. Finally, we selected ten tick species (Figure 1) which had a number of location records higher than the minimum sample size required to obtain a good performance of the MaxEnt model, i.e., 13 for widespread species [46], including *Argas persicus* (61 records), *Dermacentor marginatus* (96 records), *Dermacentor nuttalli* (65 records), *Dermacentor silvarum* (99 records), *Haemaphysalis concinna* (45 records), *Haemaphysalis longicornis* (122 records), *Ixodes granulatus* (37 records), *Rhipicephalus microplus* (41 records), *Rhipicephalus sanguineus* sensu lato (17 records) and *Rhipicephalus turanicus* (28 records). These species were reported to associate with a range of disease causative agents in mainland China, including tick-borne encephalitis virus, spotted fever group rickettsiae, Anaplasmataceae, *Borrelia burgdorferi* sensu lato, *Babesia* spp. and severe fever with thrombocytopenia syndrome virus [45,47].

### 2.2. Preparing Environmental Data

We collected bioclimatic, soil, land-use, and vegetation data from various sources to extract variables potentially important to tick survival.

Bioclimatic variables (10′) from the World Climate Database (WorldClim) were collected. The WorldClim database provided 19 bioclimatic variables commonly used in biogeographical studies [48,49], including monthly maximum temperature, monthly minimum temperature, monthly average temperature, precipitation, etc. These variables were derived from monthly meteorological records from 1970 to 2000.

Land use data (1 km) were retrieved from the Resource and Environment Data Cloud Platform of Chinese Academy of Sciences, containing several major types of land uses, i.e., cropland, forest, grassland, value shrubland, urban fabric, water surfaces, etc. These data were generated based on the classification of the Landsat images. The processing steps and quality control methods applied to these data were explained in Xu et al. [50].

Soil variables (1 km) used in the analysis were extracted from the Harmonized World Soil Database (HWSD, version 1.1, established by the Food and Agriculture Organization (FAO) of the United Nations and International Institute for Applied Systems Analysis (IIASA)), covering information on soil pH, organic carbon content, available water content, and texture.

The normalized difference vegetation index (NDVI) was downloaded from the EarthExplorer data platform of the United States Geological Survey (USGS). The NDVI is one of the most commonly used indicators to reflect the growth and nutrition condition of plants. The MODIS (the Moderate Resolution Imaging Spectroradiometer) 16 Day NDVI (250 m) datasets covering the whole of China were collected and merged.

All of these variables were rescaled on a 10′ grid (with cell size: ~340 km^2^) identical to that of the WorldClim dataset. In line with a previous study [27], the uncertainty inherent in the tick distribution data were regarded to occur on this scale. Then, all these variables were examined for multicollinearity which could cause model over-fitting. We checked correlations between each pair of the variables using the Pearson’s *r* coefficient and carefully removed variables highly correlated with the other variables (Pearson’s |*r*| > 0.8). Finally, 29 factors were screened out for further analysis and modelling (Table 1).

### 2.3. Identifying Key Factors

For each of the selected tick species, we firstly used the MaxEnt model to identify the key factors of tick distribution in China. The georeferenced tick occurrence dataset was divided randomly into two parts: 25% of the data were used to construct the model (and identify the main contributing factors) and the remaining 75% were used for model calibration. MaxEnt model is based on the second theorem of thermodynamics, and a statistical explanation on this model is detailed in Reference [51]. In the study of the potential distribution of species, the species and the environment they inhabit could be regarded as a system. A stable relationship between species and environment can be achieved when the system reached the maximum entropy, and then the contribution of different factors on the distribution of species can be estimated. We applied the same model parameter settings suggested by Raghavan et al. [27] and Alkishe et al. [10]. We used the jackknife function to identify the most important environmental factor.

In order to check the robustness of their importance in projecting tick presence, five other machine learning (ML) techniques were applied, including gradient boosting decision tree (GBDT), extremely randomized trees (ERT), random forest (RF), and the L1 and L2 support vector machines (SVM_L1 and SVM_L2). The scikit-learn software for the Python programming language was used [52,53]. A detailed explanation of these ML techniques could be found from its online user guide. The main difference among these models was that MaxEnt, GBDT, ERT, and RF took into account non-linear associations while SVM_L1 and SVM_L2 explored linear relationships between environmental factors and tick occurrence. For each tick species, the same method of random sub-setting (25% and 75% used for model construction and calibration, respectively) was applied when applying these ML algorithms.

### 2.4. Projecting Potential Tick Distribution

Potential distribution of each tick species was produced by the MaxEnt model built in the previous step. The output of MaxEnt (i.e., probability distribution of tick species) was transformed and visualized in ArcGIS software. Value 1 indicated “suitable” for ticks. A lower value indicated lower suitability for ticks, and value 0 related to “not suitable” for ticks. The performance of all the MaxEnt models of tick distribution were evaluated using the AUC (Area Under the Curve) value of the ROC (receiver operating characteristics) curve. An AUC value >0.7 is adequate; >0.8 means good; and >0.9 indicates excellent performance. Finally, the projected potential distribution was visualized in ArcGIS 10 for comparison with existing tick occurrence records.

## 3. Results

### 3.1. Environmental Determinants of Tick Occurrence

The MaxEnt model predicted that the extent of urban fabric, cropland, and forest precipitation of the driest month were the largest contributors to the modelling of the potential distribution of the ten tick species (Table 2).

(i) *Argas* ticks—For *A. persicus*, the most important environmental factors in shaping their potential habitats were the extent of urban fabric (URBAN), precipitation of the wettest month (BIO13), extents of cropland (CROP) and shrubland (SHRUB). The contribution of these factors to explaining the total variation reached 82.4%.

(ii) *Dermacentor* ticks—For *D. marginatus*, the key environmental factors were precipitation of the wettest month (BIO13), extents of cropland (CROP) and urban fabric (URBAN), minimum temperature (TMIN), pH value of the top soil (T_PH_H2O), precipitation seasonality (BIO15) and extent of grassland (GRASS), accounting for 81.0% of the total contribution to mapping tick distribution. The potential distribution of *D. nuttalli* was mainly (81.8%) influenced by the extent of urban fabric (URBAN), minimum temperature of the coldest month (BIO6), the extent of cropland (CROP), maximum temperature (TMAX), precipitation of the driest month (BIO14), isothermality (BIO3) and annual mean temperature (BIO1). The factors shaping the distribution of *D. silvarum*, the most (81.9%) were the extent of urban fabric (URBAN), monthly average temperature (TMEAN), extents of forest (FOREST) and cropland (CROP), monthly maximum temperature (TMAX), and extent of grassland (GRASS).

(iii) *Haemaphysalis* ticks—The distribution of *H. concinna* was mostly (81.7%) determined by monthly precipitation (PREC), the extent of cropland (CROP), forest (FOREST), urban fabric (URBAN), temperature seasonality (BIO4), soil available water content (AWC_CLASS) and extent of other land use types (OLU). For potential distribution of *H. longicornis* was mainly influenced by the extent of cropland (CROP), urban fabric (URBAN), annual precipitation (BIO12), annual temperature range (BIO7), monthly maximum temperature (TMAX), and extent of forest (FOREST).

(iv) *Ixodes* ticks—The key determinants of the distribution of *I. granulatus* included precipitation of the driest month (BIO14), the extent of forest (FOREST), grassland (GRASS), urban fabric (URBAN) and shrubland (SHRUB), accounting for as high as 84.3% of the total contribution.

(v) *Rhipicephalus* ticks—What shaped the potential distribution of *R. microplus* most (81.3%) were the extent of cropland (CROP), precipitation of the driest month (BIO14), minimum temperature of the coldest month (BIO6), the extent of urban fabric (URBAN) and forest (FOREST), and temperature seasonality (BIO4). For *R. sanguineus* sensu lato, the key factors (with 89.2% total contribution) were the extent of urban fabric (URBAN), monthly average temperature (TMEAN), the extent of grassland (GRASS) and annual mean temperature (BIO1). For *R. turanicus*, precipitation of the wettest month (BIO13), the extent of cropland (CROP) and urban fabric (URBAN) were found to contribute to 91.8% in predicting the distribution.

The results achieved by the other five machine learning techniques showed different degrees of agreement with the MaxEnt model. In general, the GBDT, RF and ERT models could produce similar results, by identifying the same set of land use factors as key determinants, i.e., extents of urban fabric and cropland. Besides, the GBDT, RF, and ERT predicted relatively higher contribution by precipitation of the wettest quarter and mean temperature of the coldest quarter. The support vector machines methods (SVM_L1 and SVM_L2), however, produced some different results; they tended to lower the influence of land use while stronger the climatic influence such as precipitation of the driest and the wettest month.

### 3.2. Predicted Potential Distribution of Major Tick Species

In China, the area that had relatively high suitability for tick survival was predicted to be present in North China: (i) northwest Xinjiang Uyghur Autonomous Region (XUAR) suitable for, *Argas persicus*, *Dermacentor marginatus*, and *Rhipicephalus turanicus* ticks and (ii) northeast regions including Liaoning, Jilin, Heilongjiang Provinces, and Inner Mongolia Autonomous Region where *A. persicus*, *D. marginatus*, *Dermacentor nuttalli*, *Dermacentor silvarum* and *Haemaphysalis concinna* ticks could present. Tick species could inhabit in South China, were *H. longicornis*, *I. granulatus*, *R. microplus*, and *R. sanguineus* sensu lato.

**Figure 2:**
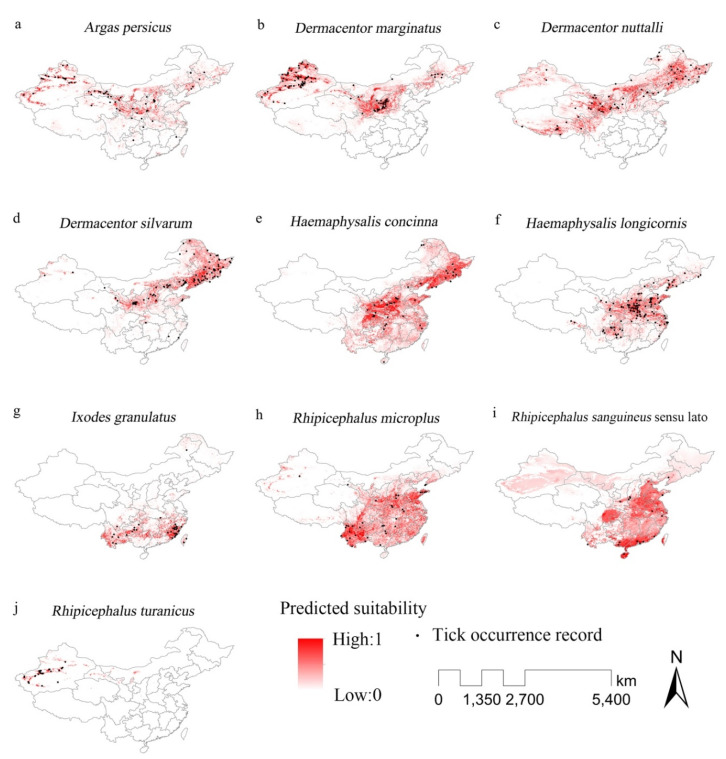
Predicted current distribution of major tick species in China. (**a**): *A. persicus*; (**b**): *D. marginatus*; (**c**): *D.* *nuttalli*; (**d**): *D. silvarum*; (**e**): *H. concinna*; (**f**): *H. longicornis*; (**g**): *I. granulatus*; (**h**): *R. microplus*; (**i**): *R. sanguineus* sensu lato; (**j**): *R. turanicus*.

(i) *Argas* ticks—The MaxEnt model predicted that *A. persicus* could establish a wide potential distribution in north China (AUC = 0.96, Figure 2a). The regions with relatively high potential of tick presence include northwest (XUAR), central north (Inner Mongolia, Shanxi, Shaanxi, Gansu, west of Henan, and Ningxia Provinces) and northeast (Liaoning, Jilin and Heilongjiang Provinces). Compared with existing tick occurrence record, the predicted distribution showed that *A. persicus* could have a wider range of distribution in central north China and in the west of XUAR.

(ii) *Dermacentor* ticks—*D. marginatus* ticks were predicted to have a potential distribution across northwest (northern XUAR), central north (Gansu, Ningxia, Shanxi, north Shaanxi, and some places in Inner Mongolia and Hebei Provinces) and northeast (north Liaoning, west Jilin, and parts of Inner Mongolia, and Heilongjiang Province) China (AUC = 0.96, Figure 2b). It should be noted that although without occurrence record, Ningxia Province was predicted to have suitable habitats for *D. marginatus*. It was predicted that *D. Nuttalli* could have an extensive potential distribution throughout the west to Northeast China (AUC = 0.93, Figure 2c), including south Gansu, Ningxia, north Shaanxi, Shanxi, Hebei, Liaoning, Jilin, Heilongjiang and Inner Mongolia. Another important region was the Qinghai–Tibet Plateau including central Tibet, west Sichuan, and parts of Qinghai and XUAR. The North China Plain (e.g., Hebei) was found suitable for *D. Nuttalli* while the existing evidence on their presence in the region was limited. *Dermacentor silvarum* also had potential habitats distributed over central north and northeast China (south Gansu, west Shaanxi, Shanxi, north Hebei, Liaoning, Jilin, Heilongjiang, Beijing and Tianjin) (AUC = 0.96, Figure 2d). Although there were some occurrence records of *D. silvarum*, South China was predicted to be less suitable for *D. silvarum*.

(iii) *Haemaphysalis* ticks—*Haemaphysalis concinna* ticks were predicted to be widely distributed in central (south Shaanxi, south Gansu, Henan, Shandong, Chongqing, east Sichuan, west Hubei, west Hunan, and south Shanxi) and northeast (Liaoning, Jilin, and Heilongjiang) China (AUC = 0.96, Figure 2e). In Liaoning Province, where occurrence records were rare, many places were found to be suitable for *H. concinna*. Compared with existing evidence, the model predicted extended west, east and south boundaries of *H. concinna*. *Haemaphysalis longicornis* ticks were predicted to mostly distributed in central to southwest China, covering Henan, Shandong, Jiangsu, Anhui, Hubei, Chongqing, north Zhejiang, south Liaoning, south Shaanxi and north Guizhou Provinces (AUC = 0.96, Figure 2f). Among these areas, Chongqing Provinces had no occurrence records of *H. longicornis* yet.

(iv) *Ixodes* ticks—The potential habitat of *I. granulatus* was predicted to be mainly in south China, including Yunnan, Guizhou, Jiangxi, Fujian, north Guangxi, south Zhejiang, and some parts of Anhui, Hunan, Guangdong, Chongqing, and Taiwan (AUC = 0.98, Figure 2g). The model predicted an extended distribution of *I. granulatus* in Jiangxi, Guangxi, Hunan, and Guangdong Provinces.

(v) *Rhipicephalus* ticks—The AUC training value of was 0.935, confirming a very good simulation result. *Rhipicephalus microplus* ticks were predicted to be able to survive largely across south China (Yunnan, Guizhou, Chongqing, Hunan, Jiangxi, Hubei, Henan, Anhui, Jiangsu, Shandong, Guangdong, east Sichuan, Fujian, Zhejiang, south Shaanxi, Guangxi, and Taiwan) (AUC = 0.94, Figure 2h). *Rhipicephalus microplus* had a predicted potential distribution far more extensive than what the existing data could tell. For example, southeast China was rarely considered as a region suitable for *R. microplus*. However, the model predicted that many places in the region had potential habitats. The potential distribution of *R. sanguineus* sensu lato ticks was predicted to cover central (south Hebei, Henan, Shandong, Jiangsu, Anhui, Hubei, and east Sichuan provinces) and south China (south Guangxi, Guangdong, Hunan, Jiangxi, Fujian, Zhejiang, Hainan and west Taiwan) (AUC = 0.93, Figure 2i). It must be pointed out that *R. sanguineus* sensu lato had a tendency of extending to the north. *R. turanicus* had a very limited potential distribution in central west XUAR (AUC = 0.99, Figure 2j). They were predicted to be likely to occur in Gansu, Inner Mongolia, and Ningxia in which no occurrence records of *R. turanicus* were found previously.

## 4. Discussion

Early MaxEnt model applications on mapping tick distribution are dated back to 2006 when the model was first established. The model has been recognized as efficient and adequate in predicting the distribution of ticks with acceptable performance [54,55]. By taking advantages of the advanced modelling method, most up-to-date tick distribution dataset in China and improved knowledge on the population ecology of ticks [28,56], this study provided novel evidence on the potential determinants and spatial pattern of tick distribution in China, where impacts of ticks were understudied. It should be noted that, the model predictions could better be understood as the environmental suitability of tick occurrence (or tick survival) rather than the real presence of tick species. The determinants could indicate that ticks were likely to find potentially suitable habitats given the cell-level land-use/soil/climatic conditions.

To our knowledge, this study was probably the first study on projecting the potential distribution of multiple major tick species for the whole of China. We found that there was an extensive amount of area in China potentially suitable for the occurrence of various tick species. Amongst the ten selected tick species, *A. persicus, D. marginatus, D. nuttalli, D. silvarum, H. concinna,* and *R. turanicus* were more likely to have suitable habitats in the relatively drier North China of the temperate continental climate. Regions with larger projected suitable tick habitats include northern Xinjiang, Liaoning, Jilin, Heilongjiang, Ningxia, Shaanxi, Shanxi, Southern Gansu, Shandong, and Henan provinces. South China, which is of tropical, subtropical monsoon climate (hot weather and ample rainfall in summer; mild weather and limited rainfall in winter) was found to have suitable habitats for *H. concinna, H. longicornis, I. granulatus, R. microplus*, and *R. sanguineus* sensu lato in Yunnan, eastern Sichuan, Guizhou, Chongqing, Fujian, Zhejiang, and Jiangsu provinces. A relevant recent study [57] predicted a similar pattern of climate suitability of *D. marginatus* presence in north Xinjiang. Finally, South Xinjiang, west Qinghai and north Tibet were projected to be less suitable for ticks to survive, owing to the fact that they belong to the Qinghai–Tibet Plateau which is known to have extreme weather conditions (long and dry winter with a strong wind as well as cold and rainy summer with hail) and low biodiversity [58].

The environmental determinants of tick occurrence identified in this study were mostly in agreement with existing findings. Among the climatic factors included, the precipitation of the driest month was found as a key determinant. Maintenance of tick population depends on tick feeding on hosts or the host-seeking activities which could be influenced by climatic factors such as relative humidity and temperature [59]. Existing studies have underlined the significant associations between questing tick pattern and precipitation [60], vapor pressure [61] and saturation deficit [62]. Specifically, *H. concinna* ticks were found to be more dependent on a humid environment, provided that precipitation and soil moisture had a greater impact on their spatial distribution.

Moreover, the MaxEnt and the five machine learning models predicted that land use played a more critical role in shaping tick distribution than climatic (temperature, precipitation), soil and vegetation (NDVI) factors considered in this study. While most existing studies [10,27,63] on projecting future tick distribution considered climatic variables solely, our findings highlighted the importance of taking into consideration future land use pattern. The extent of cropland and forests were found vital as they could provide (i) sheltered, humid microenvironment suitable for tick survival [64]; (ii) habitats for host animals which could provide blood meals essential for tick development and reproduction [65,66]. The projected distribution of selected tick species was determined by different land use factors geographically. In the center and south China, natural factors (i.e., temperature, precipitation, soil) had better contributions than in the north. The tick species in the center and south China (i.e., *H. longicornis, R. microplus*) were found to be highly associated with the extent of cropland, suggesting these species might adapt to habitats close to human agricultural activities. On the contrary, the tick species more prevalent in the north (i.e., *H. concinna, D. silvarum*) were found to be more related to forests and grassland. Such findings pinpointed the necessarily to carefully manage the likely public and veterinary health effect of forest land-use change; for example, the afforestation practices planted resulted from the giant Three-North Shelter Forest Program (1978–2050).

A very interesting finding was that the extent of urban fabric was identified as a key determinant, which seemed to be mostly neglected in studies looking into associations between tick presence and land use. Its effects were found to be different between species: the extent of urban fabric was positively related to the presence of *A. persicus, D. nuttalli, D. silvarum* and *R. sanguineus* sensu lato ticks but negatively associated with the occurrence of *D. marginatus, I. granulatus,* and *R. turanicus*. A negative association could probably mean where urban areas were dominating, tick habitats in the wild were shrunk and damaged. However, reasons for a positive association remained unclear, for which future studies are required. Nevertheless, a positive relationship between tick occurrence and extent of the urban area revealed that ticks were likely inhabited close to where people live, and, thus, a high potential risk human exposure to tick bites (and tick-borne diseases). Moreover, precipitation of driest or wettest month had greater/weaker influence on the presence of the species which were negatively/positively associated with the extent of the urban area. It thus seemed that urban land use conditions were capable of altering the effects of climatic variables on projecting tick presence.

The present study has several shortcomings, for which future improvements and research directions could be suggested. First, although the tick occurrence dataset used was the most up-to-date open-access tick data product in China, it could only support the distribution modelling of a limited number of tick species of the whole of the country. Future efforts on compiling a more comprehensive georeferenced tick dataset are therefore recommended. Second, the MaxEnt model was used since it was confirmed to have better performance in predicting the potential distribution of species with the presence-only data [67,68]. However, there are certain limitations for MaxEnt [69]; for example, it could provide a large prediction for the existing conditions beyond the study area. Future studies were suggested to make combined use of multiple modelling and evaluation approaches for coherent model projections. Third, geographical sampling bias in the tick distribution dataset was corrected to some extent by removing duplicated records in the same locations and ensuring the minimal distances between sampling records. Such corrections could lead to improvement of the model’s goodness-of-fit [70]. Future SDM studies were encouraged to conduct a careful examination and elimination of geographical sampling bias before modelling. Fourth, we only studied the potential distribution of ticks in China under the current environmental conditions. Projections of their future distribution under the plausible land-use and climatic changes would also be of importance to strengthen the prevention and control of ticks in China. Fifth, bioclimatic variables derived from remote sensing were regarded to be able to improve the prediction performance of SDM [71]. Remote sensing techniques can help to monitor the change of landscape over time [72]. Surface temperature data can be captured by remote sensing products like the Operational Land Imager (OL) on Landsat 8 and the Medium Resolution Imaging Spectrometer (MODIS), while products such as Tropical Rainfall Measurement Mission (TRMM) and Global Precipitation Mission (GPM) can provide precipitation data [35,72].

## 5. Conclusions

We conducted a modelling study to project the potential distribution of ticks—an understudied disease vector in China. By using several machine learning models, we found that the presence of urban fabric, cropland, and forest and precipitation in the driest month in a place where the key determents of tick occurrence. The MaxEnt model projected that ticks were widely distributed in the Northwest, Central, North, Northeast, and South China, with the key geographical foci being the northwest Xinjiang Uyghur Autonomous Region (for *A. persicus*, *D. marginatus*, and *R. turanicus* ticks), and northeastern regions including Liaoning, Jilin, Heilongjiang Provinces and Inner Mongolia Autonomous Region (for *A. persicus*, *D. marginatus*, *D. nuttalli*, *D. silvarum*, and *H. concinna* ticks). Future research directions are suggested towards improving the quantity and quality of the tick occurrence dataset, utilizing integrated modelling and evaluation approaches, and making future distribution projections based on both land-use and climatic conditions.

## Figures and Tables

**Table 1:** Environmental variables used in mapping tick distribution.

Symbol	Variables	Unit
BIO1	Annual Mean Temperature	℃
BIO2	Mean Diurnal Temperature Range (Mean of monthly)	℃
BIO3	Isothermality (BIO2/BIO7) (×100)	/
BIO4	Temperature Seasonality (standard deviation × 100)	/
BIO5	Maximum Temperature of the Warmest Month	℃
BIO6	Minimum Temperature of the Coldest Month	℃
BIO7	Temperature Annual Range (BIO5–BIO6)	℃
BIO10	Mean Temperature of the Warmest Quarter	℃
BIO11	Mean Temperature of the Coldest Quarter	℃
BIO12	Annual Precipitation	mm
BIO13	Precipitation of the Wettest Month	mm
BIO14	Precipitation of the Driest Month	mm
BIO15	Precipitation Seasonality (Coefficient of Variation)	/
BIO16	Precipitation of the Wettest Quarter	mm
PREC	Monthly Precipitation	mm
TMAX	Monthly Maximum Temperature	℃
TMIN	Monthly Minimum Temperature	℃
TMEAN	Monthly Mean Temperature	℃
CROP	Extent of Cropland	m^2^
FOREST	Extent of Forest	m^2^
GRASS	Extent of Grassland	m^2^
SHRUB	Extent of Shrubland	m^2^
URBAN	Extent of Urban Fabric	m^2^
OLU	Extent of other Land Use Types	m^2^
T_PH_H2O	PH Value of the Topsoil	-−Log(H^+^)
T_OC	Organic Carbon Content of the Topsoil	%weight
AWC_CLASS	Soil Available Water Content	/
T_TEXTURE	Soil Texture of the Topsoil	/
NDVI	Normalized Vegetation Index	/

**Table 2:** Key environmental variables contributed to tick distribution.

Species	Models	Environmental Variables in Order of Importance				
		1st Contributor	2nd Contributor	3rd Contributor	4th Contributor	5th Contributor
*Argas persicus*	MaxEnt	URBAN	BIO13	CROP	SHRUB	GRASS
	GBDT	URBAN	BIO13	CROP	BIO16	BIO15
	ERT	URBAN	CROP	BIO15	BIO13	FOREST
	RF	URBAN	CROP	BIO13	BIO15	BIO16
	SVM_L1	BIO13	BIO14	BIO16	URBAN	BIO7
	SVM_L2	BIO14	BIO13	URBAN	BIO5	BIO16
*Dermacentor marginatus*	MaxEnt	BIO13	CROP	URBAN	TMIN	T_PH_H2O
	GBDT	BIO13	CROP	FOREST	BIO15	T_PH_H2O
	ERT	CROP	BIO15	BIO13	OLU	BIO16
	RF	CROP	BIO15	BIO13	BIO16	URBAN
	SVM_L1	BIO14	BIO5	BIO11	BIO3	BIO13
	SVM_L2	BIO14	BIO5	BIO3	BIO13	BIO16
*Dermacentor Nuttalli*	MaxEnt	URBAN	BIO6	CROP	TMAX	BIO14
	GBDT	URBAN	CROP	TMEAN	TMAX	BIO12
	ERT	URBAN	CROP	TMEAN	BIO1	TMIN
	RF	URBAN	CROP	BIO1	TMIN	TMEAN
	SVM_L1	BIO14	BIO7	BIO4	BIO16	BIO2
	SVM_L2	BIO14	BIO7	BIO2	BIO6	BIO4
*Dermacentor silvarum*	MaxEnt	URBAN	TMEAN	FOREST	CROP	TMAX
	GBDT	URBAN	BIO1	CROP	BIO16	GRASS
	ERT	CROP	GRASS	URBAN	OLU	BIO12
	RF	URBAN	CROP	PREC	BIO12	GRASS
	SVM_L1	BIO14	BIO10	BIO1	BIO7	BIO13
	SVM_L2	BIO14	BIO10	BIO4	BIO13	BIO3
*Haemaphysalis concinna*	MaxEnt	PREC	CROP	FOREST	URBAN	BIO4
	GBDT	BIO12	FOREST	OLU	PREC	SHRUB
	ERT	FOREST	PREC	CROP	BIO12	OLU
	RF	PREC	BIO12	FOREST	SHRUB	BIO16
	SVM_L1	BIO4	BIO14	BIO7	TMAX	BIO3
	SVM_L2	BIO14	BIO4	BIO3	FOREST	BIO7
*Haemaphysalis longicornis*	MaxEnt	CROP	URBAN	BIO12	BIO7	TMAX
	GBDT	BIO6	BIO11	CROP	BIO16	URBAN
	ERT	CROP	BIO6	URBAN	BIO11	BIO7
	RF	BIO6	BIO11	CROP	URBAN	TMIN
	SVM_L1	BIO10	BIO13	BIO4	BIO14	BIO16
	SVM_L2	BIO14	BIO3	BIO13	BIO4	URBAN
*Ixodes granulatus*	MaxEnt	BIO14	FOREST	GRASS	URBAN	SHRUB
	GBDT	GRASS	BIO6	PREC	TMEAN	FOREST
	ERT	FOREST	GRASS	BIO6	BIO11	TMIN
	RF	GRASS	BIO11	BIO6	FOREST	BIO16
	SVM_L1	BIO2	OLU	BIO7	BIO14	BIO3
	SVM_L2	BIO2	OLU	BIO14	BIO3	BIO7
*Rhipicephalus microplus*	MaxEnt	CROP	BIO14	BIO6	URBAN	FOREST
	GBDT	BIO11	URBAN	TMIN	BIO14	BIO6
	ERT	BIO11	BIO6	URBAN	TMIN	BIO2
	RF	BIO6	BIO11	TMIN	URBAN	PREC
	SVM_L1	BIO4	BIO13	BIO7	URBAN	T_OC
	SVM_L2	BIO13	BIO4	URBAN	OLU	T_OC
*Rhipicephalus sanguineus* sensu lato	MaxEnt	URBAN	TMEAN	GRASS	BIO1	SHRUB
	GBDT	TMEAN	TMIN	BIO6	BIO5	SHRUB
	ERT	TMIN	CROP	TMEAN	BIO1	BIO6
	RF	TMIN	BIO1	TMEAN	SHRUB	BIO11
	SVM_L1	BIO14	BIO11	URBAN	BIO10	SHRUB
	SVM_L2	BIO14	URBAN	SHRUB	BIO6	FOREST
*Rhipicephalus turanicus*	MaxEnt	BIO13	CROP	URBAN	BIO4	AWC_CLASS
	GBDT	CROP	BIO16	BIO13	FOREST	BIO4
	ERT	CROP	OLU	BIO12	BIO16	BIO13
	RF	CROP	URBAN	BIO16	BIO13	FOREST
	SVM_L1	BIO13	PREC	BIO12	URBAN	SHRUB
	SVM_L2	BIO13	PREC	BIO12	BIO16	SHRUB
